# The Consumption of Two or Three Meals per Day with Adequate Protein Content Is Associated with Lower Risk of Physical Disability in Mexican Adults Aged 60 Years and Older

**DOI:** 10.3390/geriatrics5010001

**Published:** 2020-01-06

**Authors:** Alejandro Gaytán-González, María de Jesús Ocampo-Alfaro, Francisco Torres-Naranjo, Maritza Arroniz-Rivera, Roberto Gabriel González-Mendoza, Martha Gil-Barreiro, Juan Ricardo López-Taylor

**Affiliations:** 1 Institute of Applied Sciences for Physical Activity and Sport, Department of Human Movement Sciences, Education, Sport, Recreation, and Dance, University Health Sciences Center, University of Guadalajara, Guadalajara 44430, Jalisco, Mexico; dr.francisco.torres@icloud.com (F.T.-N.); roberto.gonzalez@academicos.udg.mx (R.G.G.-M.); taylor@cucs.udg.mx (J.R.L.-T.); 2 Department of Human Reproduction, Infantile Growth, and Development, University Health Sciences Center, University of Guadalajara, Guadalajara 44280, Jalisco, Mexico; 3 Geriatrics Department, Western General Hospital, Zapopan 45170, Jalisco, Mexico; mocampo1@prodigy.net.mx (M.d.J.O.-A.); marroniz.maritza@gmail.com (M.A.-R.); marthagilbarreiro@yahoo.com.mx (M.G.-B.); 4 Center of Body Composition and Bone Research, Guadalajara 44600, Jalisco, Mexico

**Keywords:** daily living activities, meals, physical disability, protein intake

## Abstract

Adequate protein intake per day has been associated with a lower risk of physical disability; however, if adequate protein intake per meal is also associated is unknown. The purpose of this study was to analyze the association between adequate protein intake per meal and physical disability in daily living activities in Mexican adults aged ≥60 years. We assessed the number of meals per day with an adequate protein content (24 h dietary recall), the presence of physical disability in daily living activities (two validated questionnaires), and their association in 187 participants through logistic regression. Consuming two or three meals per day with ≥30 g each was associated with lower risk of physical disability on Transportation (OR [95% CI]: 0.06 [0.01–0.50], *p* = 0.01), Shopping (0.05 [0.01–0.40], *p* = 0.004), Feeding (0.06 [0.01–0.74], *p* = 0.028), and Transfer (0.09 [0.01–0.98], *p* = 0.048). On the other hand, consuming two or three meals per day with ≥0.4 g/kg each was associated with lower risk of physical disability on Shopping (0.21 [0.05–0.89], *p* = 0.034) and Transportation (0.12 [0.03–0.48], *p* = 0.003). The consumption of two or three meals per day with adequate protein content is associated with lower risk of physical disability in Mexican adults aged 60 years and older.

## 1. Introduction

The age-related decrease in muscle mass in humans begins in the fifth decade of life [1,2,3]. This muscle mass loss often coexists with low muscle strength [4,5], functionality [6,7], and higher disability [8,9], which in turn are related with lower quality of life [10,11], and higher healthcare expenses [12], and mortality [13,14,15]. Current evidence suggests that lifestyle factors are significantly associated with functionality and physical disability. For instance, physically active older adults and those engaged in exercise programs show higher functionality and lower physical disability than their less physically active counterparts [16,17,18,19,20,21]. Additionally, dietary factors, such as low dietary protein intake, are also associated with these outcomes, whereas older adults who consume higher amounts of protein have been reported to have higher muscle mass [22,23], better functionality [24,25] and lower disability [26,27] in some studies.

Currently, it is considered that older adults should consume more protein than their younger counterparts [28,29]. Indeed, the PROT-AGE study group recommended that older adults should consume at least 1.0 to 1.2 g protein /kg body mass/day to maintain or gain muscle mass and function [28]. Similarly, 30 g protein or 0.4 g protein/kg body mass per meal (deemed adequate) are recommended to stimulate the muscle protein synthesis optimally [29,30], leading to protein accrual if it overcomes the muscle protein breakdown [31]. These recommendations for older adults are higher than for younger adults due to several age-related factors that decrease the anabolic response to amino acids in the elderly (i.e., anabolic resistance), reducing the stimulation of muscle protein synthesis and hampering the positive protein net balance [32,33]. Therefore, a higher protein intake would be needed to overcome the anabolic resistance.

In addition to daily protein intake, protein distribution, and per meal content have gained interest as other factors to consider [34,35]. Some studies have investigated if protein intake per meal at certain doses is related to muscle mass and functionality in older adults [36,37,38]. They found that the consumption of two or more meals with ≥30 g of protein each is significantly associated with higher leg muscle mass and leg strength [36], and <30 g of protein at specific meals is associated with lower functionality [37]. Nonetheless, these results have not been consistent [38].

While muscle mass, muscle strength, and functionality are strong predictors of physical disability [7,9,39], to the best of our knowledge, the approach to the association between per-meal protein intake and physical disability have not been studied in older adults. Therefore, the purpose of this study was to analyze the association between adequate protein intake per meal and physical disability on daily living activities in Mexican adults aged 60 years or older.

## 2. Materials and Methods

### 2.1. Participants

This was a cross-sectional study carried out in the Western General Hospital (Hospital General de Occidente) in Zapopan, Jalisco, Mexico. It is part of a series of articles from the same project [37]. We evaluated Mexican older adults that attended the Geriatrics Department as part of their routine medical screening or for their initial assessment from January to July 2017. Participants were eligible if: (1) they were aged 60 years or older; (2) they were able to answer to questionnaires (caregivers’ help was allowed if needed), and (3) they were able to stand up and walk unassisted (canes were permitted).

Data for analyses were excluded if participants were unable to provide detailed dietary information or if data was incomplete. This non-probabilistic sample was initially composed of 659 possible participants. However, 3 were younger than 60 years, 191 did not meet the last two inclusion criteria, 258 did not provide detailed dietary information, and 20 were discarded for missing data, leading to a final sample of 187 participants (140 women, 47 men) whose ages ranged from 60 to 97 years.

All participants gave their written informed consent before any procedure was performed. The Institutional Review Board from the University Health Sciences Center, University of Guadalajara, approved this study (CI-05518).

### 2.2. General Data

We obtained participants’ clinical information as sex, age, height (to nearest 1 cm), weight (to nearest 0.5 kg), BMI, and the number of diagnosed diseases. BMI was categorized as recommended (23–31 kg/m^2^), below (<23), and above (>31) the recommended for older adults [40].

### 2.3. Physical Disability Assessment

One researcher administrated two questionnaires to evaluate the presence of physical disability in instrumental activities of daily living (IADL) [41] and activities of daily living (ADL) [42]. Physical disability was considered as the inability or difficulty to perform daily living activities [7,9] according to the authors’ scales [41,42]. We evaluated five out of eight IADL items (those applicable for both sexes) and all ten ADL items (Table A1 in Appendix A). These two questionnaires showed good internal consistency (Cronbach’s alpha 0.94 and 0.82 for IADL and ADL questionnaires, respectively) [43,44].

### 2.4. Dietary Protein Intake Assessment

Dietary information was obtained with one 24 h dietary recall. One trained dietitian followed the USDA multistep methodology to administrate the 24 h dietary recall [45]. One external researcher calculated the total, plant, animal (all in g/day), and per meal (g at breakfast, lunch, dinner) protein intake with specialized software (Nutrickal^®^ VO v1.1, Ogali-COSINFO SC, Mexico). This external researcher has a test-retest error for estimating protein from foods ≤3.3% for all meals and total protein intake. Then, we calculated the relative protein intake per day, and per meal (g/kg/d, g/kg/meal). Next, the number of meals with a protein content ≥30 g or ≥0.4 g/kg (considered adequate [29,36]) were counted, and participants were allocated into one of three groups according to the number of meals reaching these thresholds (zero meals [0M], one meal [1M], two or three meals [+2M]). Relative protein intake per day was considered as inadequate (IPI) if <1.2 g/kg/d [28].

Finally, the coefficient of variation of protein distribution (CV) was calculated as described previously with the following equation [46]:

CV = SDP/MP(1)

where SDP is the standard deviation of the three main meals, and MP is the mean protein intake for the three main meals. The higher the CV, the more uneven the protein distribution [46].

### 2.5. Statistical Analysis

Data were assessed for normal distribution employing the Shapiro–Wilk test. If outliers were detected [47], they were excluded from that variable and analyzed again. When normal distribution was observed, we reported data as mean ± standard deviation. Otherwise, median (25th–75th percentiles) were reported. For qualitative variables, we reported them as frequencies and percentage.

We compared demographic data between included and non-included participants with χ^2^ test of independence for qualitative variables; for quantitative variables, we used *t*-test and Mann–Whitney U test for parametric and non-parametric distributions, respectively.

To compare quantitative variables between groups of meals with adequate protein intake, we used one-way ANOVA with Tukey post hoc if variables showed normal distribution and homogeneity of variances (Levene’s test). If normal distribution was observed but not homogeneity of variances, we used Welch correction and Games–Howell test as post hoc. Finally, if normal distribution and homogeneity of variances were not met, we used the Kruskal–Wallis test with Dunn’s as post hoc. For qualitative variables, we compared frequencies between groups employing the χ^2^ test of independence.

To analyze the association between the number of meals with adequate protein content and physical disability, we employed binomial logistic regression analysis, setting disability on each item as the dependent variable and the number of meals with adequate protein content as the predictive variable. The 0M group was established as the reference, and the analysis was adjusted for age (years, continuous), sex (man/woman), inadequate protein intake per day (yes/no), BMI categories, and number of diagnosed diseases (grouped as zero, one, and two or more; after reviewing their medical files looking for any physician-diagnosed disease). Data were expressed as odds ratios and 95% confidence intervals.

Additionally, we calculated the Nagelkerke pseudo R^2^ for those associations reaching statistical significance. To quantify the contribution of meals and age to the full model, we excluded one of these two variables at the time, and the pseudo R^2^ was reported. All analyses were deemed significant at a *p*-value ≤ 0.05 and were performed in SPSS^®^ v.24 (IBM Corp., Armonk, NY, USA) for Windows^®^. Graphs were elaborated with GraphPad^®^ Prism v7.05 (GraphPad Software Inc., La Jolla, CA, USA) for Windows^®^.

## 3. Results

### 3.1. Comparison between Included vs. Non-Included Participants

Non-included participants showed similar demographic characteristics and prevalence of physical disability to those observed in the included participants. None of the comparisons reached statistical significance (Table 1).

### 3.2. General and Dietary Protein Intake Characteristics (≥30 g/Meal Criterion)

When participants were classified according to the number of meals with ≥30 g of protein, 61.0% of them reported consuming zero meals, 34.2% one meal, and the remaining 4.8% two or three meals (Table 2). There were no significant differences in sex distribution, age, height, weight, BMI, BMI categories, nor the number of diagnosed diseases between these groups (Table 2). The distribution of participants by decade was as follows: 24 were aged 60–69, 81 for 70–79, 66 for 80–89, and 16 for ≥90. There were no significant differences between groups of number of meals (*p* = 0.71, data not shown).

When protein intake was assessed, IPI was significantly different between groups (Table 3). Both absolute (g/d) and relative (g/kg/d) protein intake were significantly higher for the +2M group in comparison with the other two. There were significant differences between groups for plant and animal protein intake. The CV was only different between 0 M and 1M groups (Table 3). For absolute protein intake per meal (g/meal), participants on the +2M group showed significantly higher protein intake than the other two groups for breakfast, and these differences were significant at lunch and dinner when compared with 0M group only (Table 3). For relative protein intake per meal (g/kg/meal), a similar pattern was observed (Table 3).

### 3.3. General and Dietary Protein Intake Characteristics (≥0.4 g/kg/Meal Criterion)

When participants were classified according to the number of meals with ≥0.4 g protein/kg, 41.2% of them reported consuming zero meals, 39.0% one meal, and the remaining 19.8% two or three meals (Table 4). There were no significant differences in sex distribution, age, height, nor the number of diagnosed diseases between groups. However, weight, BMI, and BMI categories differed significantly between groups (Table 4). The distribution of participants by decade showed no significant differences between groups of number of meals (*p* = 0.80, data not shown). 

When protein intake was assessed, IPI was different between groups. The absolute and relative protein intake were significantly higher for +2M group, and CV only differed between 0M and 1M groups (Table 5). There were significant differences between groups for plant and animal protein intake. For protein intake per meal, absolute intake at dinner was not different between groups, but all other meals (both absolute and relative intake) showed significant differences (Table 5).

### 3.4. Overall Physical Disabilities

About 20% of the sample reported no physical disabilities for IADL and ADL (Table 6). On the other hand, the proportion of participants reporting physical disability for all items was closer to 12% and 5% for IADL and ADL, respectively (Table 6). For IADL, the highest participants’ proportion was observed in four physical disabilities and the lowest in five, without statistical significance (*p* = 0.06). For ADL, zero physical disabilities were the most prevalent, and presenting five was less common (*p* < 0.001) (Table 6). 

### 3.5. Physical Disabilities by Group

When participants were categorized according to the number of meals with ≥30 g of protein, the most common IADL disability was observed in Shopping for 0M and 1M groups. For +2M group, Medication was the most common physical disability. There were significant differences between groups for Shopping and Transportation (Table 7). For ADL, the most commonly reported physical disability was Stairs for 0M and 1M groups. For +2M group, the most reported physical disability was on Mobility, Stairs, Dressing, and Bladder. There were significant differences between groups for Stairs only (Table 7).

When participants were categorized according to the number of meals with ≥0.4 g protein/kg, Shopping was the most common IADL disability for all groups. The proportion of participants with physical disability in Transportation and Finances differed significantly between groups (Table 8). For ADL, the most common physical disability was Stairs for all groups. There were significant differences between groups in the proportion of subjects with Stairs and Dressing disabilities (Table 8).

### 3.6. Number of Meals with ≥30 g Protein and Physical Disability

**Figure 1:**
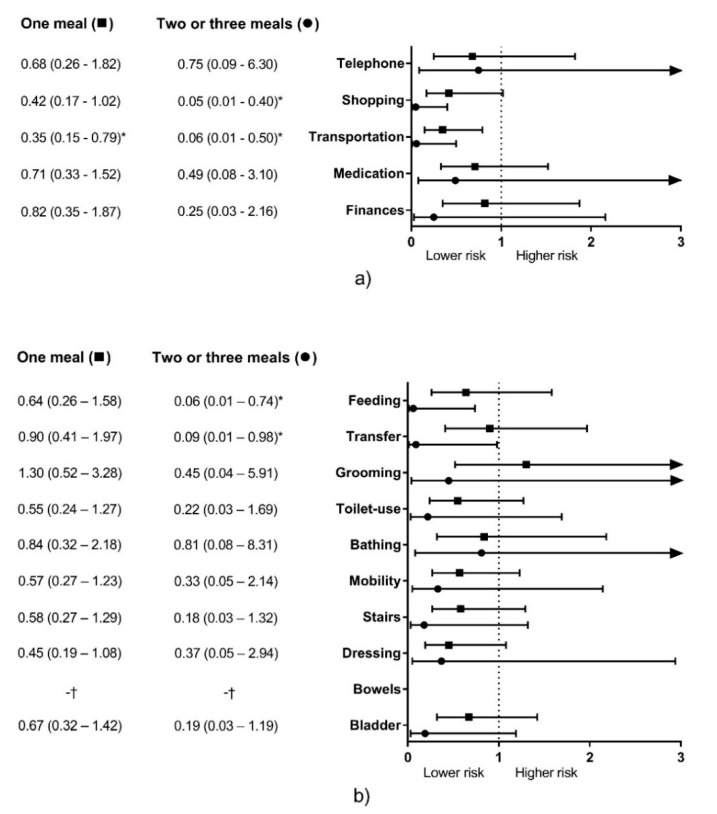
Physical disability risk on instrumental activities of daily living (**a**) and activities of daily living (**b**) depending on the number of meals per day with ≥30 g protein. Data expressed as odds ratios (95% confidence intervals), Zero meals was the reference group. The associations were adjusted for sex, age, inadequate protein intake per day, BMI categories, and number of diagnosed diseases. * Significant association (*p* ≤ 0.05). † Not calculated due to small sample size. Arrows denote 95% CI continues out of the graph scale.

For IADL, consuming 1M or +2M with ≥30 g of protein was significantly associated with lower risk of disability on Transportation (1M, *p* = 0.012; +2M, *p* = 0.01; full model R^2^ = 0.384; model excluding meals only R^2^ = 0.328; model excluding age only R^2^ = 0.167). Consuming +2M, but not 1M, was significantly associated with lower risk of disability on Shopping (*p* = 0.004; full model R^2^ = 0.315; model excluding meals only R^2^ = 0.253; model excluding age only R^2^ = 0.156). Conversely, the number of meals was not significantly associated with disability risk on Telephone, Medication, nor Finances (Figure 1a).

For ADL, consuming +2M, but not 1M, was significantly associated with lower risk of disability on Feeding (*p* = 0.028; full model R^2^ = 0.240; model excluding meals only R^2^ = 0.196; model excluding age only R^2^ = 0.143) and Transfer (*p* = 0.048; full model R^2^ = 0.287; model excluding meals only R^2^ = 0.256; model excluding age only R^2^ = 0.099). The number of meals was not significantly associated with disability risk on the other items (Figure 1b).

### 3.7. Number of Meals with ≥0.4 g Protein/kg and Physical Disability

**Figure 2:**
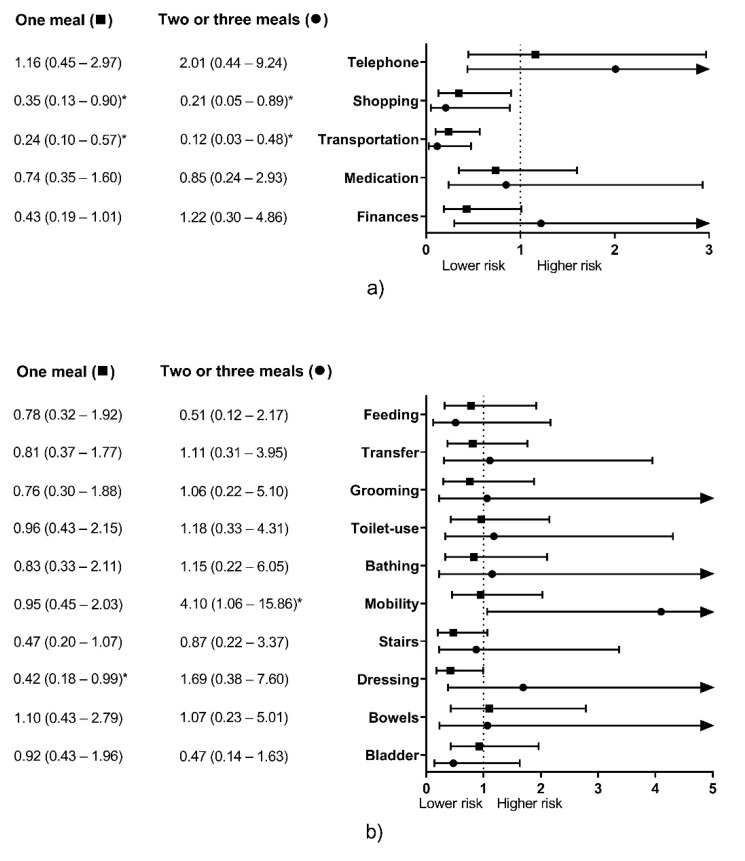
Physical disability risk on instrumental activities of daily living (**a**) and activities of daily living (**b**) depending on the number of meals per day with ≥0.4 g protein/kg. Data expressed as odds ratios (95% confidence intervals), Zero meals was the reference group. The associations were adjusted for sex, age, inadequate protein intake per day, BMI categories, and number of diagnosed diseases. * Significant association (*p* ≤ 0.05). Arrows denote 95% CI continues out of the graph scale.

For IADL, consuming 1M or +2M with ≥0.4 g of protein/kg was significantly associated with lower risk of disability on Shopping (1M, *p* = 0.03; +2M, *p* = 0.034; full model R^2^ = 0.293; model excluding meals only R^2^ = 0.253; model excluding age only R^2^ = 0.117) and on Transportation (1M, *p* = 0.001; +2M, *p* = 0.003; full model R^2^ = 0.400; model excluding meals only R^2^ = 0.328; model excluding age only R^2^ = 0.161). The number of meals was not significantly associated with disability risk on Telephone, Medication, nor Finances (Figure 2a).

For ADL, consuming 1M, but not +2M, was significantly associated with a lower disability risk on Dressing (*p* = 0.048; full model R^2^ = 0.400; model excluding meals only R^2^ = 0.363; model excluding age only R^2^ = 0.120). On the other hand, consuming +2M, but not 1M, was significantly associated with higher disability risk on Mobility (*p* = 0.041; full model R^2^ = 0.258; model excluding meals only R^2^ = 0.222; model excluding age only R^2^ = 0.081). The number of meals was not significantly associated with disability risk on the other items (Figure 2b).

## 4. Discussion

In this study, we observed that consuming two or three meals per day with ≥30 g protein each was significantly associated with lower physical disability risk for two IADL items and two ADL items. Conversely, eating one meal with this protein content was associated with one IADL item only (Figure 1).

To our knowledge, no previous studies have analyzed the association between per-meal protein intake for a certain amount and physical disability. However, there are other studies with a similar approach [36,38]. In this regard, Loenneke et al. [36] reported that older adults consuming two or more meals with ≥30 g protein showed higher leg lean mass and leg strength than those consuming one or zero meals with this criterion. We believe that consuming two or three meals with this protein content was associated with a lower risk of physical disability for more items than consuming one meal because the benefit over muscle mass and strength is bigger for two or more meals with this protein dose than with one meal [36]. Therefore, it is possible that the benefits of muscle mass and strength observed with consuming one meal are not enough to decrease the risk of disability, as happened with two or three meals.

Additionally, high protein intake is associated with a lower age-related decrease in muscle mass and muscle strength [24,48,49], and consuming more meals above this threshold is associated with higher total protein intake (Table 3). Therefore, eating two or more meals with ≥30 g of protein each might be helpful to decrease the age-related decline on lean mass and functionality to a higher level than one meal [35,50], which could lead to a lower risk of physical disability [26].

On the other hand, Gingrich et al. [38] reported that consuming two or more meals with ≥0.4 g protein/kg was not significantly associated with muscle mass, muscle strength, nor muscle power in older adults. The results of Gingrich et al. may differ from previous studies and ours because they evaluated a sample of healthier older adults (subjects with >10 points in the short physical performance battery, <5% unintended weight loss the last three months, ≈96% of the sample was well-nourished, and diabetes and impaired glucose regulation as the only clinical concerns). Indeed, the authors suggested that this kind of protein recommendation would be necessary for older adults with poorer health status.

When meals were evaluated with the ≥0.4 g protein/kg threshold, there were conflicting results. Consuming 1M and +2M containing ≥0.4 g protein/kg were significantly associated with lower physical disability risk for two IADL items, Shopping, and Transportation (Figure 2a). Conversely, consuming 1M was associated with lower physical disability risk for one ADL item (Dressing), and eating +2M was associated with higher physical disability risk for one ADL item (Mobility) (Figure 2b). Other studies also reported conflicting results with protein intake and other disability-associated variables (e.g., low skeletal muscle mass). Gregorio et al. [25] and Beasley et al. [51] reported that older adults consuming >0.8 g protein/kg/d showed lower skeletal muscle mass than those consuming protein below this threshold, and Isanejad et al. [24] reported that sarcopenic women ate more relative protein (g/kg/d) than their non-sarcopenic counterparts. It is possible that higher relative protein intake is observed in sarcopenic or disabled people because of their lower body weight [52]. However, their relative protein intake would still not be enough to stimulate muscle protein synthesis optimally. It is recommended that older people with undernutrition or another chronic illness eat more protein (1.2 to 1.5 g/kg/d) to overcome the possible anabolic resistance derived from their condition [28]. We believe that the same could happen for per-meal protein intake. Due to chronic illness may lead to inactivity and inflammation, higher protein dosage per meal could be needed to overcome the anabolic resistance and contribute to the higher daily protein intake [53].

Therefore, the amount of 30 g protein/meal could be more useful for predicting physical disability than the 0.4 g protein/kg/meal criterion in a cross-sectional design. The former significantly predicted physical disability in two items for both IADL and ADL each (Figure 1), while the latter significantly predicted disability for two IADL items, and showed conflicting results for two ADL items (Figure 2).

However, experimental studies have demonstrated that both protein dosages (i.e., 30 g/meal; 0.4 g/kg/meal) optimally stimulate muscle protein synthesis in older adults [29,30]. When muscle protein synthesis overcomes muscle protein breakdown, it leads to positive muscle protein balance, and therefore, protein accretion [31], which translates into muscle mass maintenance or increase if resistance exercise is performed [54]. Due to low muscle mass is significantly associated with weakness [5] and reduced lower limb functionality [55], the association of adequate protein intake per meal with physical disability may be mediated by its role over muscle mass [36]. Therefore, prospective studies should investigate if consuming protein above these thresholds are linked with the incidence of physical disability and its association with muscle mass.

The association between protein intake and lower physical disability risk may also be mediated by its role in decreasing body fat [26,51,56]. As we get older, there is an increase in body fat and fat infiltration into the muscle [57], which leads to decreased muscle quality, strength, and functionality [6,24,58,59]. Additionally, the lower body fat showed with high protein intake in comparison with lower protein intake [26,51], would lead to lessening the pro-inflammatory state observed in subjects with high body fat [60], which also has been reported to mediate the association between muscle strength decline and protein intake in older adults [61]. However, we did not evaluate body composition in this study. Further research should address the interaction between protein intake per meal and body composition and their association with physical disability.

These results could be helpful for the assessment of protein intake per meal in older adults, looking for those who eat less than two meals with ≥30 g or ≥0.4 g/kg as a risk factor before physical disability occurs. However, the interaction of protein intake doses with other variables, as total protein intake [23,24,25,26], physical activity level [16,17,18,19], and participation in exercise programs [19,20,21] should also be monitored in the clinical practice as risk factors.

Even though the number of meals with an adequate protein intake was a significant risk factor for physical disability (Figure 1 and Figure 2), its contribution to the model was low (3.1% to 6.9%), which could be attributable to other dietary and non-dietary factors associated with muscle mass, strength, and functionality in older adults [62,63]. Therefore, it should be considered as a significant but complementary factor when assessing proper functionality in this population. Conversely, age was the most important predictor for physical disability in this study (8% to 29.3%) in accordance with previous studies that describe the age-related decrease in muscle mass, strength, and functionality [4,10,64,65]. Hence, the role of proper dietary protein intake in older adults is, apparently, to mitigate these age-related declines [48,49,50].

It is interesting to note that adequate protein intake per meal was associated with physical disability for ambulatory items (i.e., Shopping and Transportation) in IADL, while it did not predict disability for other more cognitive-related items (Figure 1a and Figure 2a). Possibly because the role of protein over cognition is weaker than with muscle mass and physical functioning [66]. However, for ADL, Transfer was the only ambulatory item associated with adequate protein intake, and Feeding (a more cognitive-related item) was also significantly associated with adequate protein intake (Figure 1b). These differences might be attributable to how disability was considered for each questionnaire, where IADL accepts some degree of difficulty in performing daily living activities and still considers the subject is functional [41], but for ADL, the subject has to report no difficulty to be deemed functional [42].

When we analyzed the dietary protein intake, we observed that most of the protein was ingested at lunch, independently of the group or the adequate protein intake criteria (Table 3 and Table 5) which is similar to previous studies in older adults from Mexico [67], Germany [38], and France [68]. However, reports in other countries suggest that most of the daily protein is consumed at dinner [36,50,69,70,71]. Despite these country-specific differences, it is unlikely that these differences in the timing of protein intake modulate the association between the number of meals with adequate protein intake and disability. For example, Loenneke et al. [36] reported the number of meals with adequate protein intake was significantly related with strength and lean mass in American older adults (higher protein intake at dinner), while a similar result was observed here in Mexican population (higher protein intake at lunch). Nonetheless, Loenneke et al. [36] reported that 32.3% of their sample did not reach the recommended 30 g protein for any meal, and 51.8% did it for only one. In this study, we reported that 61.0% of our sample did not reach this threshold at any meal, and 34.2% did it for one only. These data let us believe that Mexican older adults would be at higher risk of presenting lower lean mass, muscle strength, and a higher risk of physical disability than that observed in American population.

There is evidence suggesting an even protein distribution throughout the day is beneficial to stimulate muscle protein synthesis optimally [72], and the protein CV has been proposed as an indicator for evaluating protein intake evenness, the lower the CV, the evener the distribution [46]. However, we observed that the CV reported in 0M and +2M groups was similar, and a higher IPI was detected in the former group for both criteria (Table 3 and Table 5). Therefore, we believe this indicator should not be used alone, as there could be an even protein distribution, but daily and per-meal protein intake might not be adequate.

We previously published data about subjects participating in the same project [37]. However, it is essential to highlight the differences between our earlier published work and the current study. While we formerly analyzed the association of timing of inadequate protein intake and functionality, we here reported that the accumulated inadequate protein intake per meal, independently of the timing, is also associated with physical disability (Figure 1 and Figure 2). Similarly, this study expands our knowledge about protein intake in Mexican older adults as we reported the dietary protein intake patterns depending on the number of meals with an adequate protein content (Table 3 and Table 5).

Although this study provides important information for the literature, some limitations must be acknowledged. Firstly, the cross-sectional design allows us to infer association between the variables, but not causality. Secondly, our data were collected by participants self-reporting over a 24 h period only and with questionnaires, which is known to lead to inaccurate data [45,73,74]. However, we followed standardized protocols and administrated validated tools to decrease this issue as possible [41,42,45]. Further efforts should be addressed to corroborate our results using more reliable and objective assessment methods.

Thirdly, our sample size was small, especially for the +2M group with the ≥30 g protein/meal criterion, where only nine participants composed this group (Table 2). This small number of participants poorly represented a portion of this population and affected the statistical analysis, which is reflected in very low odds ratios and wide confidence intervals from the logistic regression (Figure 1 and Figure 2). However, it is possible that the low proportion of participants eating adequate amounts of protein (≥30 g) for two or more meals would be similar in other samples of Mexican older adults (≈5%). Therefore, a bigger sample size must be warranted to overcome this limitation. Similarly, there were a higher number of women than men; therefore, despite the models were adjusted by sex, these results might not be representative of men. Also, our sample was recruited from a clinical setting (a regional hospital) from the west of Mexico, and might not be representative of Mexican older adults that do not attend to a medical service (due to healthier status, or inaccessibility to public healthcare) and from other geographical regions. Thus, caution must be considered when translating these results to other populations.

It is also important to highlight that despite non-included and included participants showed similar demographic characteristics (Table 1), the information about the physical disability of those unable to answer to questionnaires is lacking. Similarly, we do not know if the cognitive status could explain the inability of non-included participants to provide detailed dietary information and to answer questionnaires, as we did not measure it. Further studies should evaluate the cognitive status and use objective measures of functionality to overcome these limitations. Finally, the covariables used to adjust the models were not as varied as reported in other studies with “fully adjusted” models. However, we adjusted for basic covariables observed in most studies [23,36,48,49,51,52].

## 5. Conclusions

In our sample of Mexican adults aged 60 years and older, the consumption of two or three meals per day with ≥30 g of protein each was significantly associated with lower physical disability risk for some IADL (Shopping and Transportation) and ADL items (Feeding and Transfer). While consuming two or three meals with ≥0.4 g protein/kg was significantly associated with lower physical disability risk for two IADL items (Shopping and Transportation) only. Further research is needed to determine if these results persist in larger samples with prospective study designs.

## Figures and Tables

**Table 1:** Comparison of demographic characteristics and prevalence of physical disabilities between included and non-included participants.

	Included ^#^		Non-Included		n ^¶^	*p*
Women ^†^	140	(74.9%)	339	(71.8%)	472	0.50
Age (y) ^‡^	78.5	±7.7	78.4	±9.0	465	0.92
Height (cm) ^§^	153	(148–160)	153	(147–160)	419	0.97
Weight (kg)	62	(53–69)	63	(52–73)	429	0.72
BMI (kg/m^2^)	26.3	(22.8–29.6)	26.3	(22.8–30.5)	416	0.95
BMI category						
Below	49	(26.2%)	110	(26.4%)		
Recommended	104	(55.6%)	208	(50.0%)	416	0.29
Above	34	(18.2%)	98	(23.6%)		
Number of diagnosed diseases						
0	37	(19.8%)	77	(16.3%)		
1	77	(41.2%)	182	(38.6%)	471	0.33
≥2	73	(39.0%)	212	(45.0%)		
IADL physical disability						
Telephone	37	(19.8%)	82	(23.8%)	344	0.33
Shopping	141	(75.4%)	236	(68.6%)	344	0.11
Transportation	108	(57.8%)	211	(61.3%)	344	0.46
Medication	105	(56.1%)	191	(55.5%)	344	0.93
Finances	68	(36.4%)	140	(40.7%)	344	0.35
ADL physical disability						
Feeding	47	(25.1%)	102	(29.9%)	341	0.27
Transfer	81	(43.3%)	152	(44.6%)	341	0.78
Grooming	42	(22.5%)	77	(22.6%)	341	1.00
Toilet-use	63	(33.7%)	113	(33.1%)	341	0.92
Bathing	44	(23.5%)	84	(24.6%)	341	0.83
Mobility	90	(48.1%)	172	(50.4%)	341	0.65
Stairs	117	(62.6%)	203	(59.5%)	341	0.52
Dressing	74	(39.6%)	137	(40.2%)	341	0.93
Bowels	36	(19.3%)	65	(19.1%)	341	1.00
Bladder	94	(50.3%)	147	(43.1%)	341	0.12

^#^ n = 187. ^¶^ Due to missing data, the sample size is reported for each variable for non-included participants. ^†^ Data expressed as frequencies (percentage). ^‡^ Data expressed as mean ± standard deviation. ^§^ Data expressed as median (25th–75th percentile). ADL: Activities of daily living; BMI: Body mass index; IADL: Instrumental activities of daily living.

**Table 2:** Participants’ demographic characteristics according to the number of meals with an adequate amount of protein (≥30 g) (n = 187).

	Zero Meals		One Meal		Two or Three Meals		*p*
n	114		64		9		
Women ^†^	88	(77.2%)	48	(75.0%)	4	(44.4%)	0.09
Age (y) ^‡^	79.1	±7.4	77.4	±8.0	77.4	±9.4	0.33
Height (cm)	152.9	±8.7	154.0	±9.4	158.8	±10.4	0.16
Weight (kg) ^§^	61.8	(52.8–70.3)	63.5	(54.3–69.6)	62.0	(55.5–66.5)	0.81
BMI (kg/m^2^)	26.9	(22.6–29.8)	25.8	(23.5–28.5)	24.2	(21.3–27.0)	0.25
BMI category							
Below	33	(28.9%)	13	(20.3%)	3	(33.3%)	0.29
Recommended	58	(50.9%)	41	(64.1%)	6	(66.7%)	
Above	23	(20.2%)	10	(15.6%)	0	(0%)	
Number of diagnosed diseases							
0	24	(21.1%)	11	(17.2%)	2	(22.2%)	0.43
1	44	(38.6%)	27	(42.2%)	6	(66.7%)	
≥2	46	(40.4%)	26	(40.6%)	1	(11.1%)	

^†^ Data expressed as frequencies (percentage). ^‡^ Data expressed as mean ± standard deviation. ^§^ Data expressed as median (25th–75th percentile). BMI: Body mass index.

**Table 3:** Participants’ dietary characteristics according to the number of meals with an adequate amount of protein (≥30 g) (n = 187).

	Zero Meals		One Meal		Two or Three Meals		*p*
n	114		64		9		
IPI (<1.2 g/kg/d) ^†^	103	(90.4%)	38	(59.4%)	0	(0%)	<0.001
Protein (g/d) ^‡^	45.5	±13.5 ^a^	68.0	±11.7 ^b^	101.7	±19.3 ^c^	<0.001
Protein (g/kg/d)	0.77	±0.29 ^a^	1.11	±0.30 ^b^	1.68	±0.31 ^c^	<0.001
Plant protein (g/d) ^§^	16.0	(12.0–27.1) ^a^	19.8	(16.0–25.0) ^ab^	26.7	(17.0–32.6) ^b^	0.037
Animal protein (g/d)	28.1	(17.2–37.1) ^a^	47.4	(38.5–57.3) ^b^	74.7	(55.8–96.4) ^b^	<0.001
Animal protein (%)	62.4	(43.0–74.0) ^a^	71.3	(61.7–78.0) ^b^	72.5	(67.5–83.1) ^b^	<0.001
CV	0.45	(0.31–0.62) ^a^	0.65	(0.47–0.83) ^b^	0.48	(0.35–0.61) ^ab^	<0.001
Protein per meal (g)							
Breakfast	15.4	±6.7 ^a^	18.7	±8.6 ^b^	31.4	±9.5 ^c^	<0.001
Lunch	20.4	(12.4–25.2) ^a^	35.4	(31.1–39.2) ^b^	43.6	(34.3–50.1) ^b^	<0.001
Dinner	10.4	(6.9–14.8) ^a^	12.5	(7.9–16.7) ^ab^	34.2	(8.5–43.6) ^b^	0.017
Protein per meal (g/kg)							
Breakfast	0.24	(0.16–0.34) ^a^	0.32	(0.16–0.42) ^a^	0.47	(0.39–0.67) ^b^	<0.001
Lunch	0.31	(0.19–0.41) ^a^	0.58	(0.48–0.63) ^b^	0.74	(0.55–0.78) ^b^	<0.001
Dinner	0.16	(0.11–0.25) ^a^	0.21	(0.11–0.30) ^ab^	0.52	(0.14–0.77) ^b^	0.015

^†^ Data expressed as frequencies (percentage). ^‡^ Data expressed as mean ± standard deviation. ^§^ Data expressed as median (25th–75th percentile). Different letters denote significant differences between groups within variables (*p* ≤ 0.05). CV: Coefficient of variation of protein distribution; IPI: Inadequate protein intake per day.

**Table 4:** Participants’ demographic characteristics according to the number of meals with an adequate amount of protein (≥0.4 g/kg) (n = 187).

	Zero Meals		One Meal		Two or Three Meals		*p*
n	77		73		37		
Women ^†^	59	(76.6%)	54	(74.0%)	27	(73.0%)	0.89
Age (y) ^‡^	78.6	±7.7	77.8	±7.3	79.5	±8.6	0.54
Height (cm) ^§^	152	(147–158)	154	(148–161)	151	(149–156)	0.29
Weight (kg)	69.1	±14.2 ^a^	61.6	±10.6 ^b^	53.8	±8.4 ^c^	<0.001
BMI (kg/m^2^)	28.7	(26.2–31.8) ^a^	25.7	(22.6–27.6) ^b^	22.8	(20.8–24.8) ^c^	<0.001
BMI category							
Below	9	(11.7%)	21	(28.7%)	19	(51.4%)	<0.001
Recommended	45	(58.4%)	44	(60.3%)	16	(43.2%)	
Above	23	(29.9%)	8	(11.0%)	2	(5.4%)	
Number of diagnosed diseases							
0	17	(22.1%)	13	(17.8%)	7	(18.9%)	0.60
1	30	(39.0%)	28	(38.4%)	19	(51.4%)	
≥2	30	(39.0%)	32	(43.8%)	11	(29.7%)	

^†^ Data expressed as frequencies (percentage). ^‡^ Data expressed as mean ± standard deviation. ^§^ Data expressed as median (25th–75th percentile). Different letters denote significant differences between groups within variables (*p* ≤ 0.05). BMI: Body mass index.

**Table 5:** Participants’ dietary characteristics according to the number of meals with an adequate amount of protein (≥0.4 g/kg) (n = 187).

	Zero Meals		One Meal		Two or Three Meals		*p*
n	77		73		37		
IPI (<1.2 g/kg/d) ^†^	77	(100%)	57	(78.1%)	7	(18.9%)	<0.001
Protein (g/d) ^‡^	42.5	±13.9 ^a^	61.1	±11.7 ^b^	76.7	±19.8 ^c^	<0.001
Protein (g/kg/d)	0.62	±0.18 ^a^	1.01	±0.21 ^b^	1.43	±0.30 ^c^	<0.001
Plant protein (g/d) ^§^	16.0	(12.0–23.8) ^a^	18.0	(14.0–25.0) ^a^	24.2	(18.0–31.0) ^b^	0.001
Animal protein (g/d)	23.0	(14.4–35.9) ^a^	42.1	(31.5–48.3) ^b^	52.2	(37.2–59.5) ^b^	<0.001
Animal protein (%)	63.2	(41.0–74.2) ^a^	70.5	(60.6–77.6) ^b^	70.3	(55.4–74.2) ^ab^	0.019
CV ^§^	0.45	(0.32–0.63) ^a^	0.59	(0.41–0.82) ^b^	0.52	(0.36–0.66) ^ab^	0.006
Protein per meal (g)							
Breakfast	14.0	±6.4 ^a^	16.4	±7.3 ^a^	25.9	±7.7 ^b^	<0.001
Lunch	16.0	±8.3 ^a^	30.7	±10.5 ^b^	32.6	±10.7 ^b^	<0.001
Dinner	10.1	(6.7–14.1)	13.5	(7.9–16.1)	12.4	(6.2–22.4)	0.087
Protein per meal (g/kg)							
Breakfast	0.18	(0.14–0.30) ^a^	0.27	(0.17–0.36) ^b^	0.47	(0.41–0.54) ^c^	<0.001
Lunch	0.25	(0.13–0.33) ^a^	0.50	(0.41–0.59) ^b^	0.61	(0.48–0.74) ^b^	<0.001
Dinner	0.15	(0.10–0.19) ^a^	0.21	(0.12–0.29) ^b^	0.28	(0.12–0.44) ^b^	<0.001

^†^ Data expressed as frequencies (percentage). ^‡^ Data expressed as mean ± standard deviation. ^§^ Data expressed as median (25th–75th percentile). Different letters denote significant differences between groups within variables (*p* ≤ 0.05). CV: Coefficient of variation of protein distribution; IPI: Inadequate protein intake.

**Table 6:** Participants’ distribution depending on the number of physical disabilities reported in activities of daily living (ADL) or instrumental activities of daily living (IADL) (n = 187).

Number of Disabilities	IADL		ADL ^a^	
	n	(%)	n	(%)
0	36	(19.2)	38	(20.3)
1	30	(16.0)	27	(14.5)
2	25	(13.4)	23	(12.3)
3	28	(15.0)	18	(9.6)
4	45	(24.1)	16	(8.6)
5	23	(12.3)	6	(3.2)
6	-		15	(8.0)
7	-		8	(4.3)
8	-		12	(6.4)
9	-		15	(8.0)
10	-		9	(4.8)

^a^ Distributions significantly different within ADL (*p* ≤ 0.05). Calculated with χ^2^ goodness of fit.

**Table 7:** Frequency and percentage of participants with physical disability depending on the daily living activity and number of meals with adequate protein intake (≥30 g).

	Zero Meals (n = 114)		One Meal (n = 64)		Two or Three Meals (n = 9)		*p* *
	n	(%)	n	(%)	n	(%)	
IADL							
Telephone	25	(21.9)	10	(15.6)	2	(22.2)	0.59
Shopping	95	(83.3)	43	(67.2)	3	(33.3)	0.001
Transportation	75	(65.8)	30	(46.9)	3	(33.3)	0.016
Medication	67	(58.8)	33	(51.6)	5	(55.6)	0.65
Finances	45	(39.5)	21	(32.8)	2	(22.2)	0.45
ADL							
Feeding	29	(25.4)	17	(26.6)	1	(11.1)	0.60
Transfer	53	(46.5)	27	(42.2)	1	(11.1)	0.12
Grooming	27	(23.7)	14	(21.9)	1	(11.1)	0.68
Toilet-use	43	(37.7)	18	(28.1)	2	(22.2)	0.33
Bathing	29	(25.4)	13	(20.3)	2	(22.2)	0.74
Mobility	62	(54.4)	25	(39.1)	3	(33.3)	0.10
Stairs	80	(70.2)	34	(53.1)	3	(33.3)	0.014
Dressing	51	(44.7)	20	(31.3)	3	(33.3)	0.20
Bowels	24	(21.1)	12	(18.8)	0	(0)	0.30
Bladder	62	(54.4)	29	(45.3)	3	(33.3)	0.30

ADL: Activities of daily living; IADL: Instrumental activities of daily living. * *p*-value obtained from χ*^2^*test of independence.

**Table 8:** Frequency and percentage of participants with physical disability depending on the daily living activity and number of meals with adequate protein intake (≥0.4 g/kg).

	Zero Meals (n = 77)		One Meal (n = 73)		Two or Three Meals (n = 37)		*p* *
	n	(%)	n	(%)	n	(%)	
IADL							
Telephone	14	(18.2)	13	(17.8)	10	(27.0)	0.47
Shopping	64	(83.1)	51	(69.9)	26	(70.3)	0.12
Transportation	53	(68.8)	35	(47.9)	20	(54.1)	0.031
Medication	44	(57.1)	37	(50.7)	24	(64.9)	0.36
Finances	32	(41.6)	18	(24.7)	18	(48.6)	0.022
ADL							
Feeding	17	(22.1)	17	(23.3)	13	(35.1)	0.29
Transfer	33	(42.9)	29	(39.7)	19	(51.4)	0.51
Grooming	19	(24.7)	14	(19.2)	9	(24.3)	0.69
Toilet-use	24	(31.2)	23	(31.5)	16	(43.2)	0.39
Bathing	19	(24.7)	15	(20.5)	10	(27.0)	0.72
Mobility	37	(48.1)	30	(41.1)	23	(62.2)	0.11
Stairs	55	(71.4)	38	(52.1)	24	(64.9)	0.047
Dressing	34	(44.2)	20	(27.4)	20	(54.1)	0.015
Bowels	14	(18.2)	14	(19.2)	8	(21.6)	0.91
Bladder	39	(50.6)	36	(49.3)	19	(51.4)	0.98

ADL: Activities of daily living; IADL: Instrumental activities of daily living. * *p*-value obtained from χ^2^ test of independence.

## Appendix A

**Table A1:** Items’ definitions for instrumental activities of daily living (IADL) and activities of daily living (ADL).

Scale	Item	Definition ^a^
IADL	Telephone	The ability to use the telephone independently (dialing and/or answering).
	Shopping	The ability to go shopping independently.
	Transportation	The ability to drive his or her own car, using public transportation, or a taxi independently.
	Medication	The ability to take its own medicine in proper doses and moments independently.
	Finances	The ability to handle daily living purchases and budgets independently.
ADL	Feeding	The ability of proper self-eating, including cutting foods.
	Transfer	The ability to stand-up from a chair and moving to the bed independently.
	Grooming	The ability to handle personal hygiene.
	Toilet-use	The ability to reach the toilet, undressing properly, wiping, dressing, and leaving independently.
	Bathing	The ability of proper self-care while taking a shower.
	Mobility	The ability to move or walking independently (sticks or canes are allowed).
	Stairs	The ability to ascend and descend stairs.
	Dressing	The ability to select and put on clothes independently.
	Bowels	The ability to control the bowels.
	Bladder	The ability to control the bladder.

^a^ Definitions were modified from the authors’ original redaction. For a more in-depth explanation, see [41,42].
